# Low Metabolic Variation in Environmentally Diverse Natural Populations of Temperate Lime Trees (*Tilia cordata*)

**DOI:** 10.3390/metabo15080509

**Published:** 2025-07-31

**Authors:** Carl Barker, Paul Ashton, Matthew P. Davey

**Affiliations:** 1 Department of Biology, Edge Hill University, Ormskirk L39 4QP, UK; crl.barker@googlemail.com; 2 Scottish Association for Marine Science (SAMS), Oban PA37 1JQ, UK

**Keywords:** ecology, *Tilia*, metabolic variation, functional traits, specific leaf area

## Abstract

**Background**: Population persistence for organisms to survive in a world with a rapidly changing climate will require either dispersal to suitable areas, evolutionary adaptation to altered conditions and/or sufficient phenotypic plasticity to withstand it. Given the slow growth and geographically isolated populations of many tree species, there is a high likelihood of local adaption or the acclimation of functional traits in these populations across the UK. **Objectives**: Given the slow growth and often isolated populations of *Tilia cordata* (lime tree), we hypothesised that there is a high likelihood of local adaptation or the acclimation of metabolic traits in these populations across the UK. Our aim was to test if the functional metabolomic traits of *Tilia cordata* (lime tree), collected in situ from natural populations, varied within and between populations and to compare this to neutral allele variation in the population. **Methods**: We used a metabolic fingerprinting approach to obtain a snapshot of the metabolic status of leaves collected from *T*. *cordata* from six populations across the UK. Environmental metadata, longer-term functional traits (specific leaf area) and neutral allelic variation in the population were also measured to assess the plastic capacity and local adaptation of the species. **Results**: The metabolic fingerprints derived from leaf material collected and fixed in situ from individuals in six populations of *T*. *cordata* across its UK range were similar, despite contrasting environmental conditions during sampling. Neutral allele frequencies showed almost no significant group structure, indicating low differentiation between populations. The specific leaf area did vary between sites. **Conclusions**: The low metabolic variation between UK populations of *T*. *cordata* despite contrasting environmental conditions during sampling indicates high levels of phenotypic plasticity.

## 1. Introduction

Population persistence for organisms to survive in a world with a rapidly changing climate will require either dispersal to suitable areas, evolutionary adaptation to altered conditions and/or sufficient phenotypic plasticity to withstand them [1,2,3,4]. Although many species have moved recently in response to current changes [5,6], it is likely that the rate of change outstrips the dispersal ability of many others [7,8]. If rapid dispersal is not an option, as in the case of slow-growing trees, then populations must possess sufficient phenotypic plasticity to withstand changes in the short term and enough adaptive variation to allow for evolutionary responses in the long term [9,10,11]. Plasticity can therefore buffer against rapid change but may also assist in evolutionary adaptation via selection for stronger plastic responses [3,12,13]. Given this, some tree species might be well-placed and locally adapted to respond rapidly to environmental change [14].

Such local adaptation is less likely in species with small or fragmented ranges, as selection is less efficient due to the increased effects of drift on any given locus [15]. For example, *Tilia cordata* Mill. (Small leaved Lime tree) is a widespread temperate canopy tree that possesses a fragmented distribution, often occurring in small and isolated populations. Although it possesses high levels of neutral genetic variation and low levels of genetic differentiation [16,17], these cannot be used as evidence of either the presence of adaptive variation or a lack of local adaptation, respectively, as neutral and adaptive variation are only weakly related [18,19]. Given the significant reductions in precipitation that are projected for the Mediterranean [20], both declines in abundance and local extinctions have been predicted in Italian populations [21]. Therefore, it is important to understand both how *T. cordata* will respond to environmental changes in the short term (via phenotypic plasticity), and to assess the likelihood of evolutionary change in threatened populations [22].

Common garden experiments that examine the genetic basis of phenotypic traits, such as the specific leaf area [23,24], are a typical method of establishing the presence of local adaptation in plant species (‘provenance trials’) and therefore the existence of adaptive variation among populations [14]. Gene sequencing or population genomics approaches can indicate the presence of local selection [25], but this approach requires foreknowledge of the traits of interest [26]. A metabolomic trait analysis approach [27], where the concentration or presence of some of these metabolites, can represent the ultimate response of biological systems to genetic or environmental changes [26,28]. Consequently, it has been used to understand plant–environment interactions [29,30], as well as to detect signatures of local adaptation in natural populations [31,32,33,34,35,36]. Unfortunately, *T. cordata* is not an experimentally tractable species [37,38,39], which makes common garden, reciprocal transplant or glasshouse approaches difficult. Therefore, an exploratory approach characterizing metabolic variation in situ within and between populations using metabolomic techniques is required [28]. As the scale at which metabolic variation was detectable in previous work is large (hundreds of kilometres), an appreciable geographic range needs to be sampled [34].

To provide additional information, neutral genetic and adaptive morphological variation can also be assessed, as stochastic processes such as drift affect both neutral and functional genetic variation [40]. If differences in metabolite fingerprints are observed and they have a genetic basis, but differentiation across selectively neutral molecular markers is not found, then this would suggest local selection. Additionally, the metabolome is the product of many factors, including daily fluctuating metabolic processes and the current environmental context [41,42]. As a result, it is a ‘snapshot’ of processes occurring at a variety of temporal scales [43]. In contrast with this, functional traits such as the specific leaf area (SLA) are largely influenced by longer term climatic conditions [44,45,46] and have been shown to be adaptive and exhibit inter-population variation [23].

Given the slow growth and often geographically isolated populations of *T. cordata*, as discussed above, there is a high likelihood of local adaptation or the acclimation of metabolic traits in these populations across the UK. As such, we would hypothesise and expect to be able to measure these metabolic differences collected in situ from natural populations in the UK. To this end, we assessed if a portion of the metabolome of *T. cordata*, collected in situ from natural populations, varied within and between populations and if this variation related to relevant laboratory, environmental metadata or to the site of origin (i.e., can signals of metabolic differences be detected when sampling in-situ). The aim is also to explore the possibility of using metabolic fingerprints from field-collected samples in a hypothesis-generating role to allow for assessments on both the plastic capacity and local adaptation of the species.

## 2. Materials and Methods

### 2.1. Sampling Strategy and Locations

**Figure 1:**
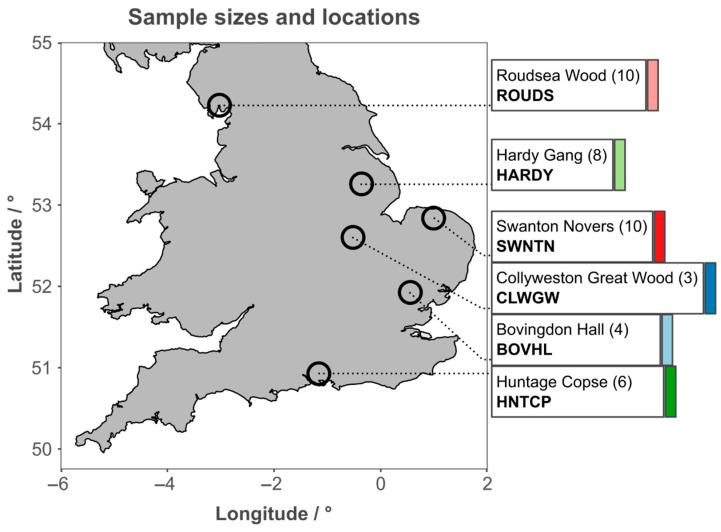
The geographic location in the UK of all sampling sites, along with the number of samples per population. Open circles indicate the mean position of samples, while boxes connected by a dotted line to these indicate the respective name, sample size (in brackets), and abbreviation used for each. Coloured bars indicate the colour used to refer to each site in all further figures.

Six populations of *T. cordata* spread across its UK range were sampled (Figure 1) [16,22]. To minimize variation in environmental conditions, the sampling within sites was standardized as far as possible. The data are also provided in the PhD Thesis by CB at https://research.edgehill.ac.uk/en/studentTheses/ecological-genetic-and-metabolic-variation-in-populations-of-tili (accessed on 15 July 2025).

East-facing trees that received similar insolation were located, and the specific individuals to be sampled were chosen randomly. To reduce differences in ontogeny, the sampling dates (as day length) were kept as similar as practicably possible. From each tree, four branches spread across the canopy that received full sunlight were collected. Branches with obvious signs of pathogen damage or herbivory were avoided. The terminal leaves of these were cored with a metal leaf corer (four cores per leaf), pooled and placed in dry-shippers charged with liquid nitrogen before being stored in the laboratory at −80 °C. To examine whether SLA differed between sites, the fourth and fifth leaves of each sampled branch were removed and placed in a plastic bag containing moistened tissue to avoid moisture loss and stored at 4 °C.

To explore the contribution of sampling methods and laboratory procedures to the metabolic variation, relevant ‘metadata’ during both the collection and analysis of each sample was recorded [47]. As metabolite concentrations can show temporal variability [41], the time of day was noted and converted to hours since dawn based on latitude and date using the R software (version 3.3.3; R Core Team, 2016) with the package maptools, version 0.8-41 [48]. Other factors likely to affect primary metabolism via changes in the photosynthetic rate or transpiration were also measured: wind speed and ambient temperature were recorded with a handheld anemometer; relative humidity was noted with a handheld hygrometer; and photosynthetically active radiation (PAR) reaching each sample was recorded using a handheld photometer.

### 2.2. Metabolite Extraction

The four cores from each individual leaf were split into pairs and extracted, run and analysed separately as biological replicates, to allow for an assessment of the variability within individuals. Metabolites were extracted using a similar procedure to Davey et al. [31]. Each leaf pair was weighed, ground in a mortar and pestle with sterile sand, and transferred to a 2 mL microcentrifuge tube. A mixture containing methanol, chloroform and water (2.5:1:1) was added (40 µL mg^−1^ leaf tissue), vortexed to mix and then left on ice for 30 min. Tubes were centrifuged at 16,000× *g* for 2 m and the supernatant was transferred to a fresh 15 mL centrifuge tube (so separating the liquid from the sand and remaining leaf tissue) and stored on ice. A mixture of methanol and chloroform (1:1) was added to the pellet (20 µL mg^−1^), vortexed to mix, and the tube was stored on ice for a further 30 min. This was then centrifuged as before and the supernatant transferred to the 15 mL tube.

This extract was separated into nonpolar, chloroform and aqueous methanol phases by the addition of distilled water at 4 °C (10 µL mg^−1^) and centrifugation at 16,000× *g* for 1 min. Then, 300 µL of the polar phase was transferred to glass vials and stored at −80 °C until analysis. Both solvent mixtures were chilled to −20 °C before use, and the extraction procedure was undertaken in small, randomly chosen groups (two to three pairs) in a randomized order. These sequences were also retained as metadata to assess if the sample handling introduced any variation between samples. Extraction blanks were generated by repeating this procedure three times without any leaf material.

### 2.3. Metabolite Fingerprinting

The metabolic fingerprints of the polar metabolites present in the aqueous phase were generated using a Synapt G2 (Waters Ltd., Elstree, UK) matrix-assisted laser desorption/ionization time-of-flight mass-spectrometer (MALDI-TOF MS) in both positive and negative ion modes. Samples were combined 1:1 with a 5 mg mL^−1^ α-Cyano-4-hydroxycinnamic acid (CHCA) matrix and ionized with a solid-state Nd:YAG UV laser (355 nm). Spectra were collected by direct injection mass spectrometry in the mass range of 100–800 Da at a rate of one spectrum s^−1^ (1 s scan time, 0.02 s inter-scan delay).

In positive mode, the mass spectrometer operated with an accelerating voltage of 10 V, an 11 V hexapole bias and an aperture voltage of 7 V. The negative-mode settings were identical, except for a lower hexapole bias and aperture voltage (10 V and 5 V, respectively). The raw spectra of mass/charge ratios were processed as described in [31]. The putative annotation of metabolites was performed based on a comparison of detected masses with the KEGG database [49]. Spectra were rounded into 0.2 Dalton bins and the relative abundance of each was used to produce profiles of the percent total positive and negative ion content for each replicate (%TIC).

Pareto-scaling (division of each variable by the square root of its standard deviation) was applied to both positive and negative %TIC datasets as it reduces the relative importance of large values but keeps the structure of the data intact [50,51]. To avoid the generation of nonsensical data, several invariant bins with a standard deviation of zero had to be removed prior to scaling (Table S1). The bins representing the CHCA matrix used in the ionization process were also removed from both datasets before analysis (Table S1) as these signals are also detected by the instrument and can obscure biological information [52].

### 2.4. Collection of Functional Trait Data

The specific leaf area (SLA) was recorded following Cornelissen et al. [45]. The one-sided leaf area was measured using an AM350 Area Meter (ADC BioScientific Ltd., Hoddesdon, UK), and then the leaves were oven-dried at 80 °C for 48 h and weighed individually. The SLA was calculated for each individual as the mean of each of its leaves’ area divided by their mass (10 n for each tree).

To compare the SLA between sites, the normality and homoscedasticity of collected data was first assessed using Shapiro–Wilk and Bartlett tests, respectively [53]. This indicated that the data were normal, but that the groups were significantly heteroscedastic. Therefore, a nonparametric Kruskal–Wallis hypothesis test was applied and where required, a Bonferroni-corrected Dunn’s multiple comparison test was used to ascertain where significant differences occurred between the groups [54].

### 2.5. Assessment of Neutral Genetic Variation

For a comparison of potentially adaptive variation and neutral genetic variation, all individuals were genotyped across 10 microsatellite loci using the ‘crude extract’ procedure described in Barker [55]. Allele frequencies at these loci are in Hardy-Weinberg equilibrium in a variety of populations of *T. cordata* [17]. These markers are therefore not expected to be under selective pressure.

### 2.6. Multivariate Data Exploration

Principal components analysis (PCA) was applied to both positive and negative %TIC datasets using the R package vegan, version 2.4-2 [56]. This was performed both with and without extraction blanks to assess the level of variation induced by technical issues such as instrumental noise [50]. To explore whether the environmental context or laboratory methods were related to the observed variation in metabolite concentrations, all metadata were used to colour-code plots displaying the first two principal components (PC).

As PCA summarises the axes of greatest variation through the data, it only reveals the group structure when the within-group variation is less than the between-group variation [27]. In contrast, partial least squares-discriminant analysis PLS-DA reduces the dimensionality of the data by producing latent variables that are linear combinations of the original variables that maximise covariance between response and class membership data (here site of origin) [52,57]. Given this approach it can find separation between groups where none exists and so should be used in concert with PCA to guide interpretation [52]. Therefore, PLS-DA was also applied to both positive and negative %TIC datasets using the R package mixOmics, version 6.2.0 [58]. An assessment of how well these models discriminate between groups was performed using leave-one-out cross-validation (LOOCV), which generates new PLS-DA models by excluding one observation across all observations and uses these to predict the class membership for each excluded observation [59]. This allows for the calculation of error rates, i.e., what proportion of individuals are misclassified.

The genetic data were explored in a comparable way. As population genetics studies typically have higher sample sizes than used here, the relationship of genetic distance (1-percentage shared alleles [DPS]) [60]) with geographic location was assessed using distance-based redundancy analysis (dbRDA) [61,62]. If enough individuals have been genotyped, then the populations can be expected to exhibit isolation-by-distance (IBD), as observed previously, given the spatial scale of the study. PCA was then applied to allele frequency data for comparison with the ordinations of metabolite concentration. All data analysis was undertaken in the R package vegan, version 2.4-2 [56].

## 3. Results

### 3.1. Metabolic Variation

**Figure 2:**
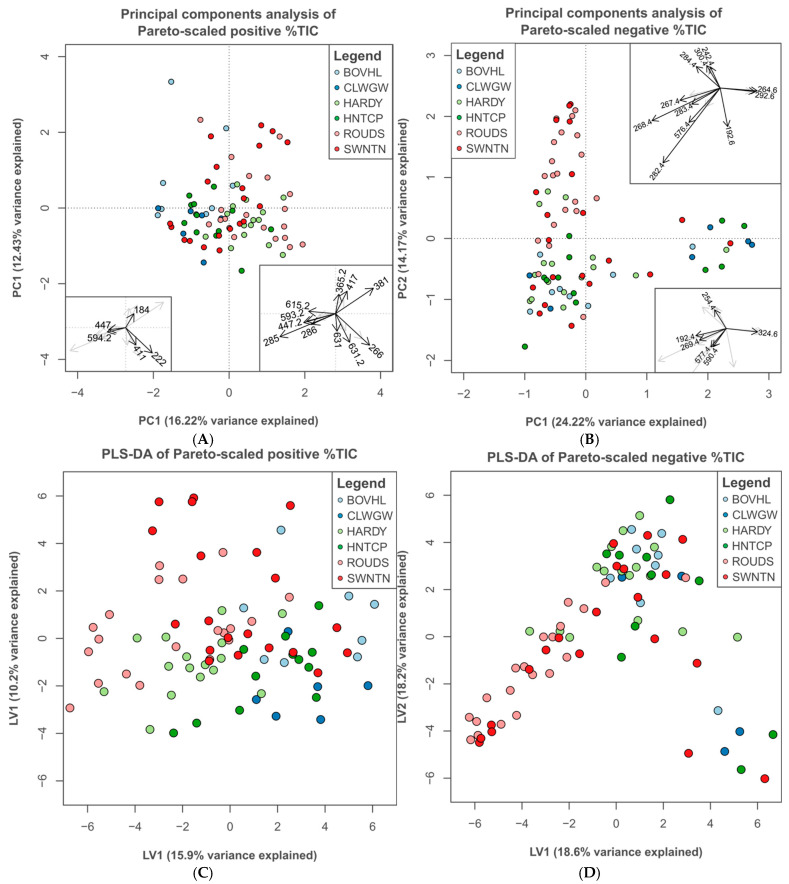
Principal components analysis (PCA) of percent total ion content (%TIC) in all samples analysed in (**A**) positive and (**B**) negative ionisation mode. Only the first two components (PC1/PC2) are shown. Colour indicates site of origin. Insets show variable loadings for each of the two components, illustrating the most influential mass bins in the analysis; only the ten highest absolute loadings for each component are labelled with their respective mass bin size. Partial least squares-discriminant analysis (PLS-DA) of Pareto-scaled total ion content (%TIC) of samples analysed in positive (**C**) and negative (**D**) ionisation mode. Only two latent variables were generated for this model.

Both positive and negative-mode TOF mass spectrometry revealed variations in metabolite concentrations across sites, as illustrated by the averaged %TIC within each molecular mass bin (Figure S1). The first two components of PCA for both datasets summarise an acceptable proportion of the variation in %TIC between samples (positive: 28.65%; negative: 38.39%), given the extremely high dimensionality of the data (3493 and 3499 variables, respectively) (Figure 2). Variation between sites was low, as illustrated by the large overlap in the ordination of the first two PCs in samples analysed in the positive mode (Figure 2A). In the negative dataset, however, all sites bar ROUDS contained individuals that received similar scores along PC1 and appear together in a separate cluster, while the remaining individuals primarily differ by scores received on PC2 (Figure 2B). The sites ROUDS and SWNTN had the greatest variation along this axis, and several individuals from each were distinct from the other groups as a result. Scores received by biological replicates in both ordinations revealed that there was intra-individual variation.

Supervised ordinations of positive and negative %TIC profiles had similar patterns to the unsupervised PCA. The two latent variables (LV1/LV2) of PLS-DA for the positive dataset illustrate the lack of variation between sites, with distinctly overlapping groups (Figure 2C). A similar structure to the PCA was present in the PLS-DA of negative %TIC (Figure 2D). Most individuals vary along one direction, with the sites ROUDS and SWNTN exhibiting the most variation and the former group the most distinct. Based on leave-on-out cross-validation, the overall error rates for both PLS-DA models were high (>60%; Figure S2).

Each PC is composed of contributions from all mass bins, but the relative importance of each varies. Due to the high number of variables within the data, only the ten highest loadings for each component of both ordinations are provided. The mass bins with the highest contributions to principal components derived from positive %TIC profiles range from 184 to 632.2 Da (Table 1), and many are strongly correlated with each other, as indicated by the narrow angle between loading arrows (Figure 2 insets). The ten highest ranked loadings from the negative PCA covered a similar but slightly narrower range (192.4–590.4 Da), and the majority of important bins were between 254.4 and 324.6 Da (Table 2). Comparisons of the metabolite masses detected with the KEGG database suggested many potential candidates for the identity of compounds in the positive %TIC profiles (Table S2). In most bins, there was no clear pattern to the putative annotations, with metabolites involved in a wide array of metabolic pathways (Table S2), but the size matches in bins 381 (PC1: 2nd, PC2: 3rd) and 365.2 (PC2: 7th) were primarily oligosaccharides.

The mass bins with the highest contributions to the latent variables of PLS-DA derived from positive %TIC profiles ranged from 184 to 647.2 Da, a very similar range to high-ranking loadings in the equivalent PCA (Figure S3). The mass bins most important to the PLS-DA of negative %TIC profiles range from 192.6 to 592.4 Da, again similar to that of the PCA. There was a comparable pattern in the masses of important variables, with the majority of high-ranking bins being between 242.4 and 300.4 Da. Overall, the supervised ordinations did not provide more or distinct information compared to the unsupervised examples.

### 3.2. Environmental Metadata

**Figure 3:**
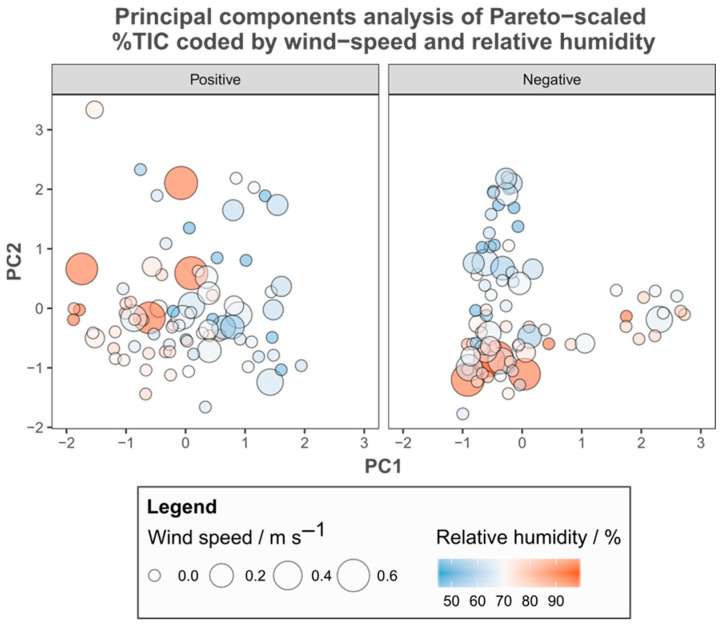
Principal components analysis of Pareto-scaled positive and negative percent total ion content (%TIC), coded by metadata indicating the environmental context at time of sampling. Individuals are represented by filled circles whose colour indicates the relative humidity while the size of these circles reflects the recorded wind speed.

**Figure 4:**
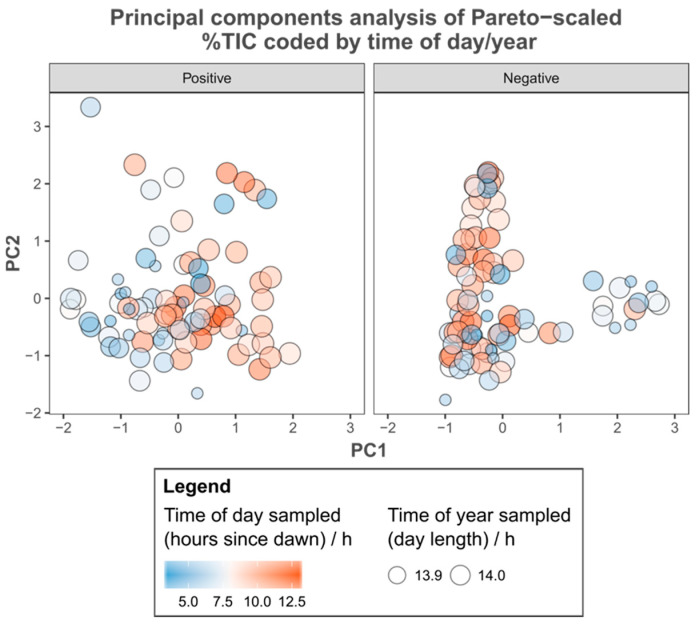
Principal components analysis of Pareto-scaled positive and negative percent total ion content (%TIC), coded by temporal metadata. Individuals are represented by filled circles whose colour indicates the time of the day (as hours of daylight since dawn) they were sampled. The size of these circles reflects the sampling date (as day length).

**Figure 5:**
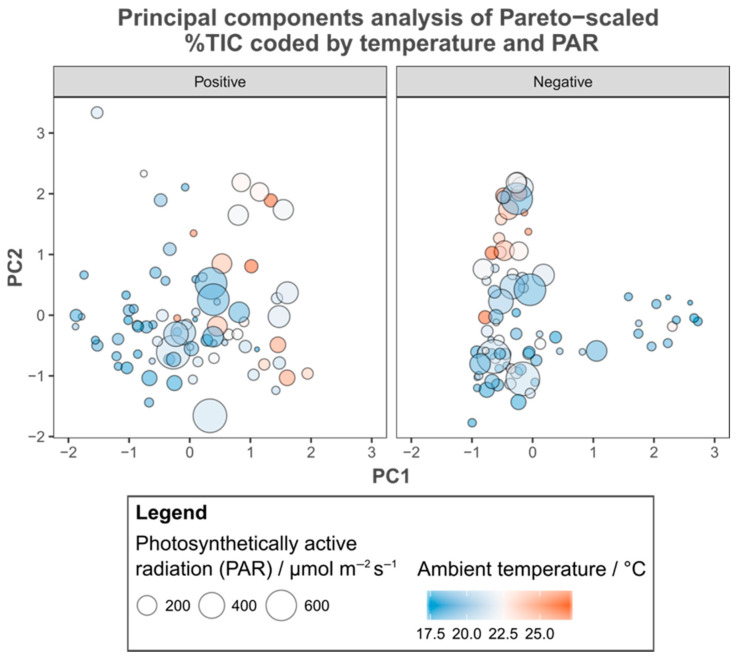
Principal components analysis of Pareto-scaled positive and negative percent total ion content (%TIC), coded by metadata indicating the environmental context at time of sampling. Individuals are represented by filled circles whose colour indicates the ambient air temperature while the size of these circles reflects the amount of photosynthetically active radiation potentially received.

A comparison of the metadata recorded with PCA ordinations derived from both positive and negative %TIC revealed few apparent relationships. Wind speed (Figure 3), hours since dawn, time of year (Figure 4), order of extraction on the bench and the time spent in storage before analysis (Figure S4) varied independently of all axes examined. Individuals sampled with high relative humidity cluster in the negative %TIC PCA (Figure 3). However, in a comparison with group membership, these patterns represent site of origin (i.e., individuals sampled at the same time in the same environmental context share similar metadata), rather than being related to the variance by the PCs. The amount of photosynthetically active radiation (PAR) recorded was related to the variance summarised by the first PC of the ordination derived from positive %TIC across groups (Figure 5).

### 3.3. Neutral Trait Genetic Variation

**Figure 6:**
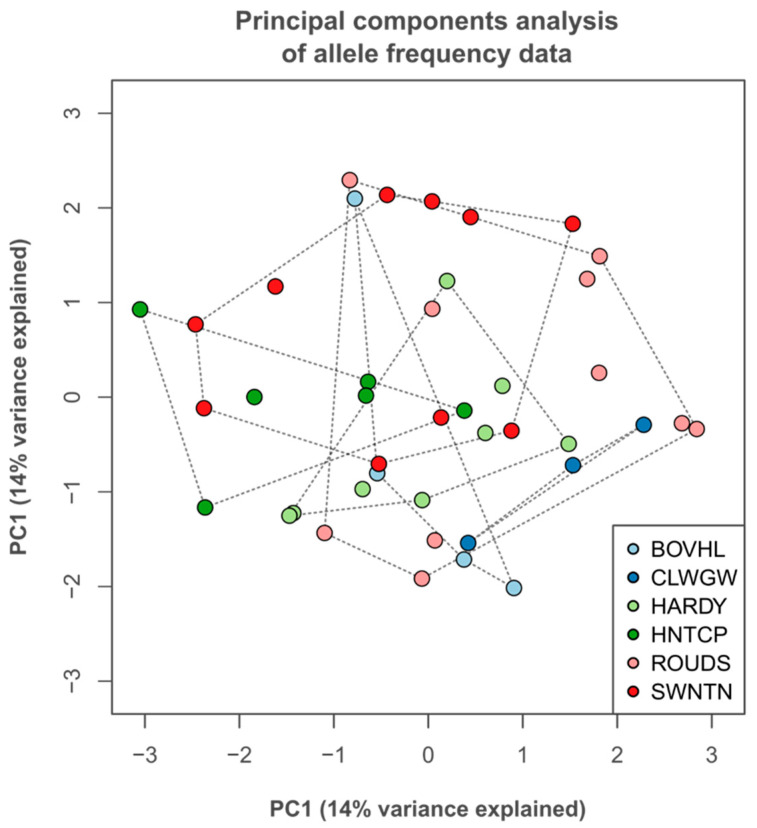
Principal components analysis (PCA) of microsatellite allele frequency data for all sampled individuals. Only the first two components (PC1/PC2) are shown. Filled coloured circles represent the scores received by each individual for each of these axes. Site groups are linked by a dashed line.

An examination of neutral genetic variation produced similar results to that of metabolic variation, with the differentiation between groups being low. The genetic distance (proportion of shared alleles) was, however, significantly but weakly related to the geographic distance between individuals (R^2^ = 0.054; *F* = 2.25, df. = 1, *p* = 0.013), indicating the occurrence of isolation by distance. Low differentiation is reflected in a PCA of allele frequencies, which exhibits no group structure (Figure 6).

### 3.4. Functional Trait Specific Leaf Area Variation

**Figure 7:**
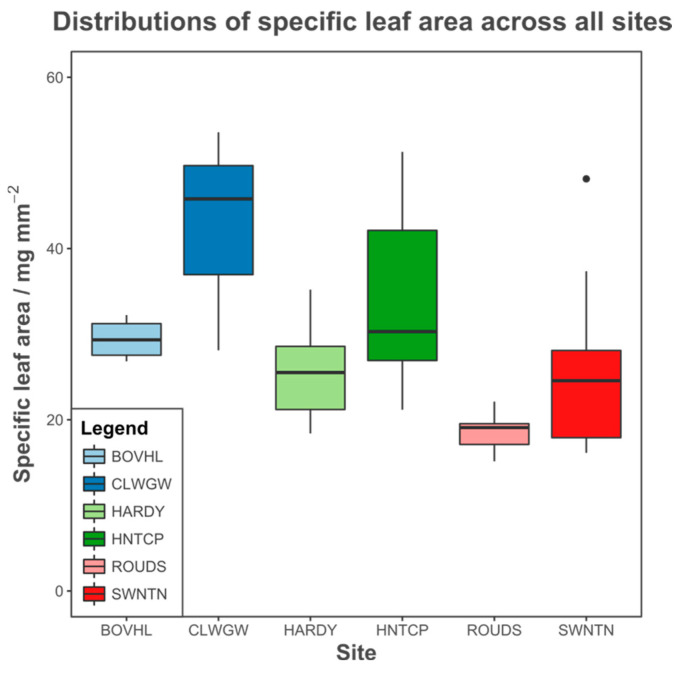
Box-and-whisker plot of specific leaf area (SLA: one-sided leaf area/leaf dry mass) for all sampled individuals across all sites. Whiskers represent 1.5 × the inter-quartile range (IQR). Any observations greater than 1.5 × IQR are represented as a dot.

In contrast to the low variation in functional metabolic %TIC profiles and neutral genetic variation, the measured specific leaf area (SLA) functional trait was distinctly different between locations (Kruskal–Wallis H = 19.384, df. = 5, *p* < 0.01). A Bonferroni-corrected Dunn’s test for multiple comparisons indicated that this result was generated by a significantly different median SLA at ROUDS versus all other sites bar HARDY (Table S3). The variation in SLA was also lower in ROUDS than at other locations (Figure 7).

## 4. Discussion

Metabolomics approaches have been used to detect the signatures of local adaption to distinct selective pressures, as well as to elucidate plant–environment interactions [28,31,43], both of which have implications for population persistence in response to rapid environmental change [11,63]. Here an exploratory approach to characterizing the in situ metabolic and morphological variation within and between populations was used. This variation was also contrasted with neutral genetic variation derived from microsatellite markers.

Metabolic fingerprints derived from leaf material collected and fixed in situ from individuals in six populations of *T. cordata* across its UK range were similar despite contrasting environmental conditions during sampling. Both unsupervised (PCA) and supervised (PLS-DA) ordinations revealed little group structure, illustrating that most of the variation in metabolite concentrations summarised by these techniques occurred across rather than between populations. These analyses summarised datasets with extremely high dimensionality (~3500 variables), reducing them down to two variables that described an appreciable amount of the variance in the overall datasets. Populations exhibited considerable overlap and hence plasticity and this was reflected in the high overall error rates in the classification performed by PLS-DA (>60%).

Putative metabolites annotations were made based on their detected masses. Searches of the KEGG database provided many candidates for the most important positive %TIC mass bins, but only two for those of negative %TIC. These loadings are positive, and so individuals with higher scores along these axes possess higher concentrations of simple carbohydrates. All of the potential identifications made for positive %TIC bins should be interpreted with caution due to the nature of the ionization process used. More specifically the charged metabolites detected during MALDI-TOF spectrometry are composed of the compound itself (M) and the addition or removal other ions (H^+^, Na^+^, K^+^).

In contrast, there was a distinct pattern to most loadings of negative %TIC ordinations, as indicated by the relatively narrow mass range of the highest-ranking bins. However, given the greater precision of detected masses for these metabolites few candidate compounds were returned from the KEGG database. Only the bins 576.4 and 269.4 resulted in matches, with phosophoribosyl-formimino-AICAR-phosphate (PRFAR) and estrone, respectively, being suggested. PRFAR is in intermediate in histidine biosynthesis, and histidine, being a proteinogenic amino acid, is involved in many aspects of plant metabolism [64]. Estrone is an exclusively mammalian hormone and so this identification is likely in error [65], but many plant compounds in the flavonoid group have similar molecular weights and structures (e.g., isoflavones) [66]. It is possible that the patterns present in both PCA and PLS-DA of negative %TIC are created primarily by differences in the concentration of this class of secondary metabolites, several of which have already been observed in the species previously [67]. These compounds also often occur in leaf material as glycosides (i.e., with the addition of a sugar molecule), bringing their molecular weight into the 500–600 Da range, similar to the more massive high-ranking bins. If concentrations of secondary metabolites are indeed generating these structures then the lack of putative identifications is not surprising, since although primary metabolites are often conserved between taxa and are therefore easily identified, the majority of secondary products are genus or even species specific [68].

A comparison of the recorded metadata with the output of PCA suggested that the relative humidity at the time of sampling may have been related to metabolite concentrations, but comparing group identity with these apparent patterns shows that they reflect similarity due to spatial and temporal proximity (i.e., individuals at BOVHL; cf. Figure 2B). In other words, individuals from the same site possess similar environmental metadata, but not other individuals with similar metabolic fingerprints, indicating that neither of these two factors were responsible. Similarly, neither wind speed nor time of sampling (either time of day or year) were related to the variation summarised by PCA. More importantly none of the laboratory metadata were related either, which alongside distinct extraction blanks illustrates that the results reflect the actual variation in metabolic status at the time of sampling rather than induced variation as a result of technical issues [50]. However, incident PAR appeared related to the first principal component derived from positive %TIC, which is not unexpected given that this is a key determinant of photosynthetic rate [69,70,71].

Both the lack of an obvious relationship between metadata and metabolite concentrations and the diversity of metabolic roles played by flavonoids [72] make it difficult to comment on what other external environmental factors might be driving the variation summarised by ordinations of negative %TIC. For example, variation in flavonoid concentration can be caused by an array of processes, including biotic and abiotic stressors such as temperature, herbivory, plant–pathogen interactions, drought and nutrient deprivation [73,74,75,76]. Overall, metabolic fingerprints are the result of a dense network of biosynthesis routes [77] and so the plastic portion of the variance examined here is likely generated by a variety of environmental factors. However, it is important to clarify that there are limitations in the level and type of metabolites that can be detected in this fingerprinting method. It is indeed a fact that, at this analytical level, there were no significant detectable differences (and so we conclude that the detectable metabolic signal is highly plastic), but we do expect detectable differences given the right equipment and enough temporal sampling points over day and night.

Allele frequencies produced similar patterns to metabolite concentrations, i.e., no group structure, indicating low differentiation between populations (little between-site variation). The inter-individual distance was, however, weakly related to geographic location, indicating the presence of weak IBD. This is in line with previously observed results [17,55], which shows that the sample size was sufficient to detect genetic structure. Conversely, leaf morphology was distinct between sites, with SLA lower at ROUDS than all other more southerly sites bar HARDY. Along with BOVHL, it also possesses low variability relative to the other groups. ROUDS experiences the lowest average temperature while BOVHL experiences both the highest average temperature and the lowest level of precipitation of all sampled sites [78]. Since both the temperature and moisture regime affect SLA, these results are consistent with the observation that abiotic filtering is stronger in certain environments. For instance, a lower leaf area per unit mass, as well as reduced variability in the same, has been observed along both latitudinal and altitudinal gradients of temperature [24,44,79,80,81].

Whether differences in SLA and variation in (potential) secondary metabolite concentrations represent responses to local selection or phenotypic plasticity cannot be determined here. Both however have been observed to be components of local adaptation in other tree species. For instance more drought-tolerant *Fagus sylvatica* individuals in Spanish populations had higher flavonoid concentrations than more northern populations when grown in a common garden setting [82], and similarly, differentiation in SLA was linked to drought-tolerance in populations of *Quercus suber* [23]. Temperature has also been observed to be a cause of local selective differences that generate metabolic differentiation between populations, e.g., changes in nitrogen metabolism in *Arabidopsis lyrata* along a latitudinal gradient [31]. Stochastic processes (e.g., genetic drift, founder effect) can also cause chemical differentiation [40], but these should affect neutral genetic variation too. Given the low genetic differentiation observed, any of the observed differences between populations (variation in putatively-identified flavonoid concentration, SLA changes) can be expected to be the result of local selective pressures provided they have a genetic basis.

## 5. Conclusions

The original hypothesis of this study was that the metabolic traits in leaves from isolated populations of *T*. *cordata* across the UK would be highly distinguishable between populations given their likelihood of local adaptation. In addition we tested whether the metabolic traits and specific leaf area co-varied with other environmental metadata. This would allow the possibility of using metabolic fingerprints in field-collected samples to explore in a hypothesis-generating role to allow for both assessments on the plastic capacity and local adaptation of the species. Our findings show the high-level plasticity of *T. cordata*, as it was able to maintain very similar metabolic profiles within distinct environmental settings. Even if the distinctions made between populations here do not reflect the presence of adaptive variation, this phenotypic plasticity may assist population persistence in the future [1,3,13].

This study also shows that metabolite profiles derived from samples collected in situ from natural populations have potential in a hypothesis-generating role, as does non-targeted metabolite profiling generally [68]. For instance, now that a functional trait difference between populations has been established, it should be examined further, to determine if it has a genetic basis, by either common garden or glasshouse experiments or more realistically, given the potential logistical difficulties of such an approach in *T. cordata*, a targeted genetic study (e.g., sequencing of homologous genes linked to SLA in other species) [83,84].

## Figures and Tables

**Table 1:** Ten highest absolute loadings for each principal component derived from positive-ionisation percent total ion content (%TIC). Where possible, putative identifications from the KEGG database [49] are shown along with their respective metabolic pathways.

Mode	Component	Mass Bin	Rank	Loading	Putative Identification	Amino Acid Metabolism	Synthesis of Secondary Metabolites	Cell Signalling	Cell Membrane Metabolism	Carbohydrate Metabolism	Citric Acid Cycle	Purine Metabolism	Pyrimidine Metabolism
Positive	PC1	285	1	−0.42	Various (10)	✓	✓	✓	✓	✓	✓		
		381	2	0.29	Oligosaccharides; galactinol; panthetheine 4′-PO_4_	✓	✓	✓	✓	✓	✓		
		266	3	0.26	Amino acids (3), nucleosides (2)	✓	✓	✓	✓	✓	✓		✓
		447.2	4	−0.24	Various (10)	✓	✓	✓	✓	✓	✓		
		593.2	5	−0.22	-								
		615.2	6	−0.20	-								
		222	7	0.20	Amino acids (5), amino sugars (3)	✓	✓	✓	✓	✓	✓		
		286	8	−0.18	Pyridoxal-PO_4_; pyrodoxine-PO_4_; linamarin	✓	✓	✓	✓	✓	✓		
		594.2	9	−0.14	-								
		447	10	−0.13	CDP-ethanolamine; Khellol glucoside				✓				
	PC2	266	1	−0.27	Amino acids (3), nucleosides (2)	✓	✓	✓	✓	✓	✓		✓
		631.2	2	−0.25	Diosmin; reserpine								
		381	3	0.22	Oligosaccharides; galactinol; panthetheine 4′-PO_4_	✓	✓	✓	✓	✓	✓		
		222	4	−0.22	Amino acids (5), amino sugars (3)	✓	✓	✓	✓	✓	✓		
		285	5	−0.20	Various (10)	✓	✓	✓	✓	✓	✓		
		417	6	0.19	-								
		365.2	7	0.16	Oligosaccharides; XMP; GA44; galactinol; ajmaline	✓	✓	✓	✓	✓	✓	✓	
		631	8	−0.15	-								
		411	9	−0.15	2′-Deoxyuridine 5′-diphosphate								✓
		184	10	0.12	Amino acids (4); selenophosphate	✓	✓	✓	✓	✓	✓		

✓ denotes putatively identified metabolite is present in that pathway

**Table 2:** Ten highest absolute loadings for each principal component derived from negative-ionisation percent total ion content (%TIC). Where possible, putative identifications from the KEGG database [49] are shown along with their respective metabolic pathways are provided.

Mode	Component	Mass Bin	Rank	Loading	Putative Identification	Amino Acid Metabolism	Synthesis of Secondary Metabolites	Cell Signalling	Cell Membrane Metabolism	Carbohydrate Metabolism	Citric Acid Cycle	Purine Metabolism	Pyrimidine Metabolism
Negative	PC1	268.4	1	−0.32	-								
		282.4	2	−0.27	-								
		267.4	3	−0.19	-								
		264.6	4	0.17	-								
		292.6	5	0.16	-								
		324.6	6	0.15	-								
		576.4	7	−0.15	Phosphoribosyl-formimino-AICAR-phosphate	✓	✓						
		192.4	8	−0.14	-								
		283.4	9	−0.13	-								
		269.4	10	−0.13	Estrone			✓					
	PC2	282.4	1	−0.46	-								
		192.6	2	−0.24	-								
		576.4	3	−0.21	Phosphoribosyl-formimino-AICAR-phosphate	✓	✓						
		268.4	4	−0.20	-								
		300.4	5	0.14	-								
		284.4	6	0.13	-								
		242.4	7	0.13	-								
		254.4	8	0.11	-								
		577.4	9	−0.11	-								
		590.4	10	−0.11	-								

✓ denotes putatively identified metabolite is present in that pathway

## Data Availability

The original contributions presented in this study are included in the article/supplementary material. Further inquiries can be directed to the corresponding author(s). The data are also provided in the PhD Thesis by CB at https://research.edgehill.ac.uk/en/studentTheses/ecological-genetic-and-metabolic-variation-in-populations-of-tili (accessed on 15 July 2025).

## Supplementary Materials

The following supporting information can be downloaded at: https://www.mdpi.com/article/10.3390/metabo15080509/s1. Supplementary Table S1. Data bins removed from each metabolite concentration (percent total ion content, %TIC) dataset prior to analysis and the rationale behind removal. Supplementary Table S2. Putative identifications for the ten highest absolute ranking mass bins in principal components analysis. Supplementary Table S3. Bonferroni-corrected Dunn’s multiple comparison test of specific leaf area between all pairwise combinations of sites. Supplementary Figure S1. Site-wide means of percent total ion content (%TIC) for each mass bin across all individuals and biological replicates within each site. Supplementary Figure S2. Error rate of the partial least square-discriminant analysis (PLS-DA) models. Supplementary Figure S3. Variable loadings plots from the PLS-DA. Supplementary Figure S4. Principal components analysis of Pareto-scaled positive and negative percent total ion content (%TIC), coded by metadata describing laboratory procedure.

## Abbreviations

Da (Dalton); DIMS (Direct Injection Mass Spectrometry); PCA (Principal Component Analysis); %TIC (Percent Total Ion Count); SLA (Specific Leaf Area).
