# Supplementary material for: Low Metabolic Variation in Environmentally Diverse Natural Populations of Temperate Lime Trees (Tilia cordata)
Source: Metabolites. 2025 Jul 31;15(8):509. doi: 10.3390/metabo15080509 (PMC12388547; doi:10.3390/metabo15080509)
Supplement: Supplementary file 1 [file metabolites-15-00509-s001.zip › metabolites-3684668-supplementary.pdf]

Supplementary data

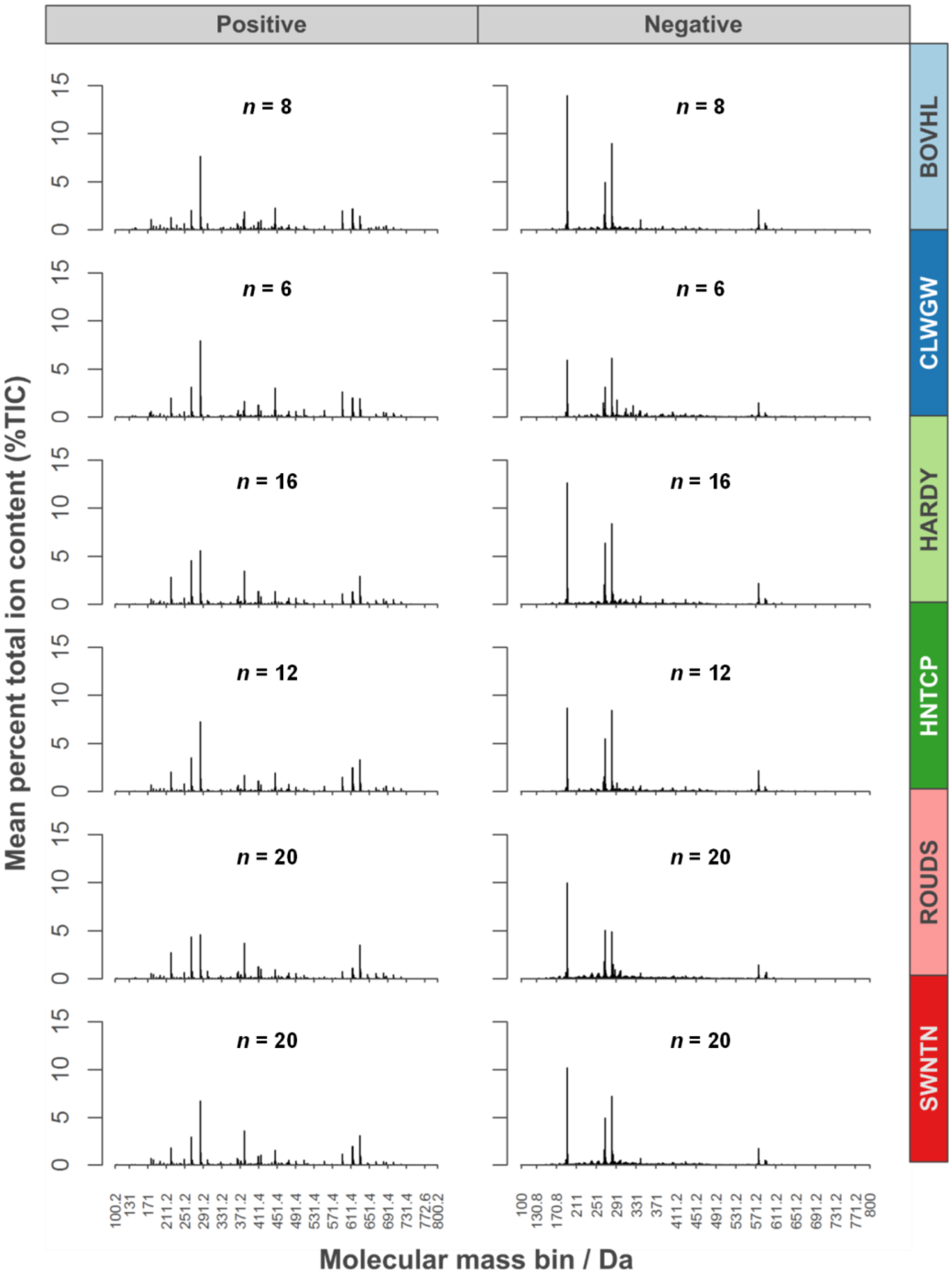

Figure S1: Site-wide means of percent total ion content (%TIC) for each mass bin across all individuals and biological replicates within each site. Left-hand charts contains %TIC for metabolites detected in positive mode while charts on the right contain %TIC for those detected in negative mode. Site codes are given on the right:. BOVHL (light blue): Bovingdon Hall; CLWGW (dark blue): Collyweston Great Wood; HARDY (light green): Hardy Gang; HNTCP (dark green): Huntage Copse; ROUDS (pink): Roudsea Wood; SWNTN (red): Swanton Novers.

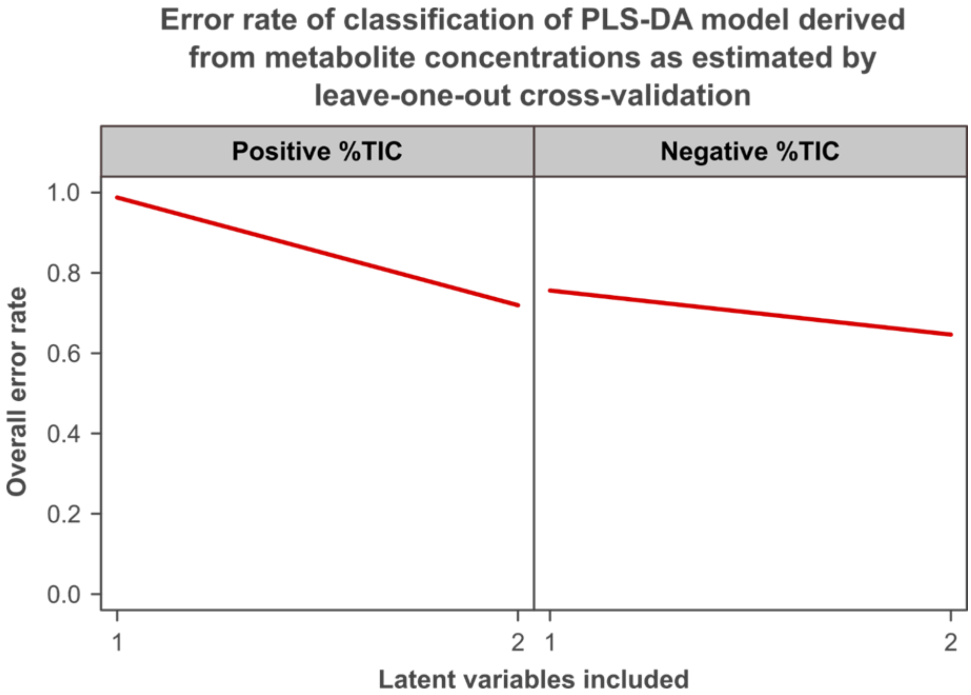

Figure S2: Error rate (i.e. what proportion of individuals are misclassified across all groups) of the partial least square-discriminant analysis (PLS-DA) models for both positive and negative %TIC.

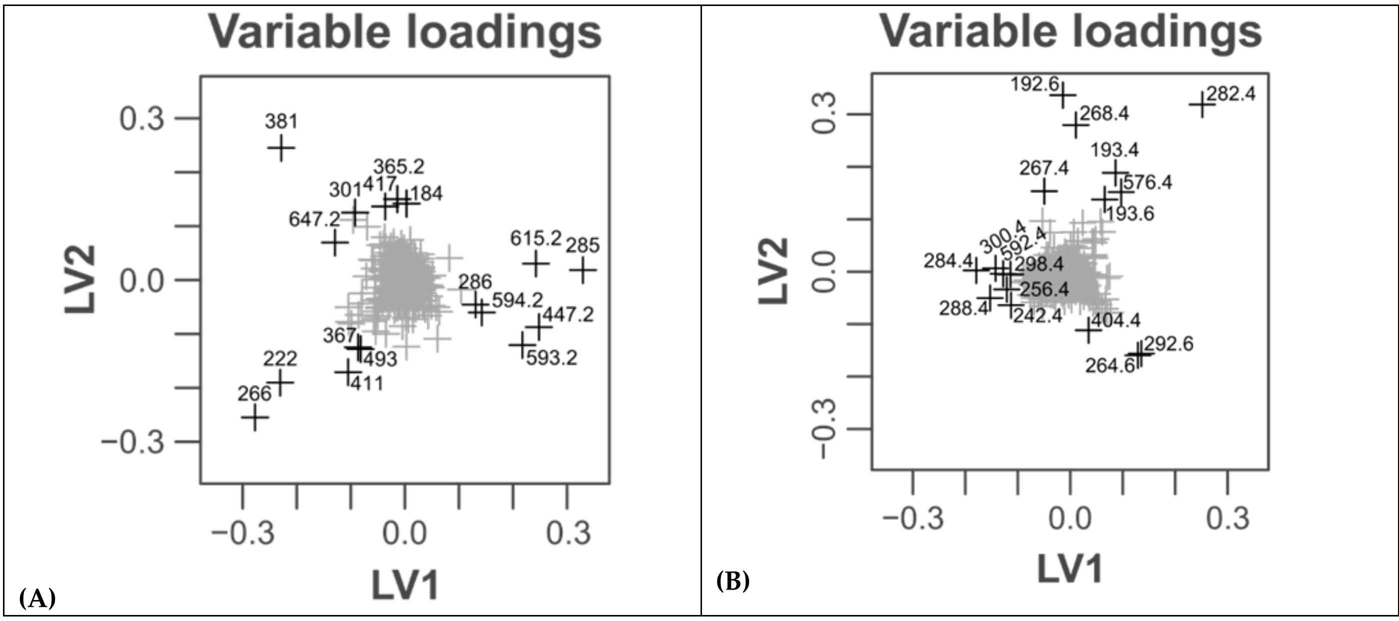

Figure S3: Variable loadings plots from the PLS-DA for samples analysed in positive (**A**) and negative (**B**) ionisation mode. For the sake of clarity only the ten highest absolute loadings for each latent variable are labelled.

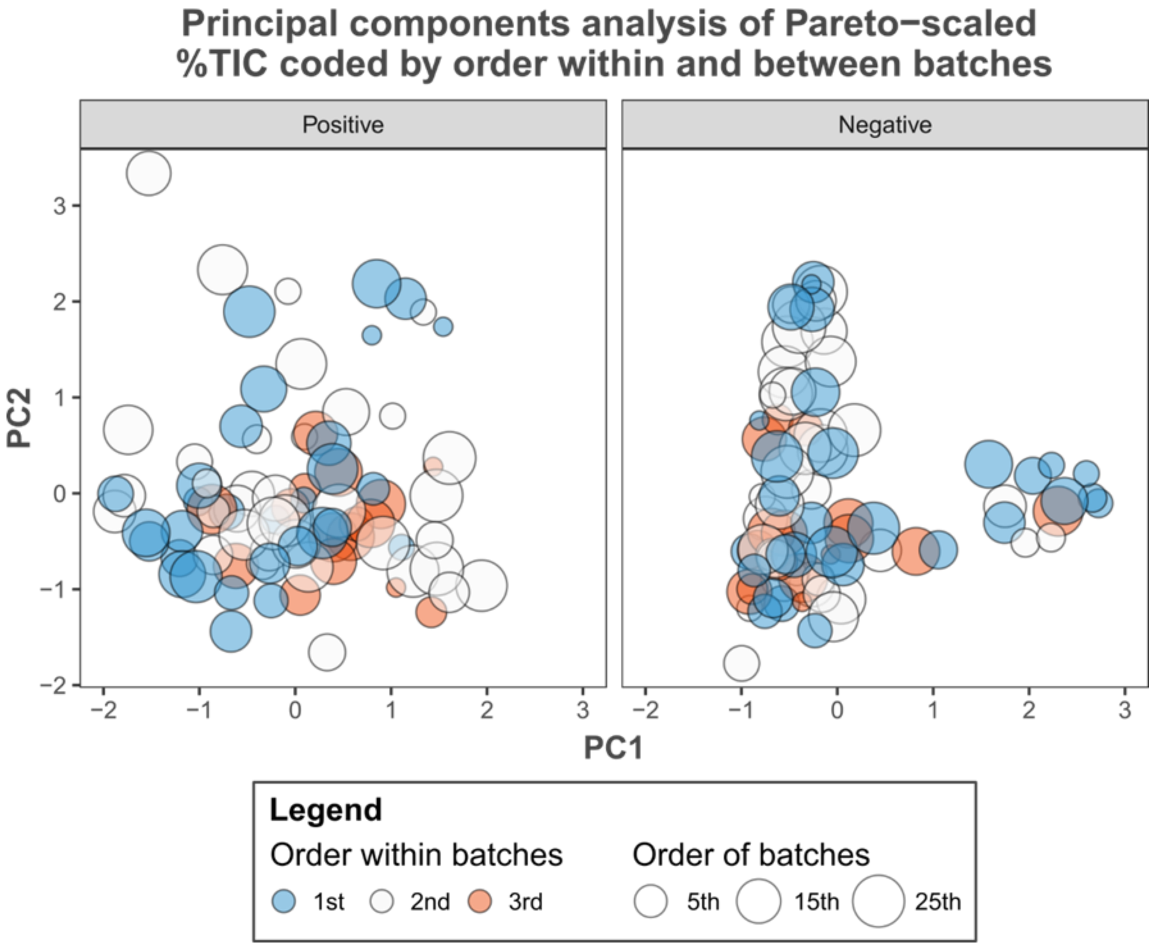

Figure S4: Principal components analysis of Pareto-scaled positive and negative percent total ion content (%TIC), coded by metadata describing laboratory procedure. Individuals are represented by filled circles whose colour indicates the order in which samples were processed on the bench, while the size of these circles reflects the overall order of metabolite extraction.

Supplementary Table S1: Data bins removed from each metabolite concentration (percent total ion content, %TIC) dataset prior to analysis and the rationale behind removal.

	Data removed		Rationale
	Positive %TIC	Negative %TIC	
Mass bin	172.0	172.0	Matrix bins
	379.0	379.0	
	100.0	800.2	Invariant
	752.8		
	753.0		
	753.2		
	753.4		
	753.6		
	765.8		
	800.4		
	800.6		

Supplementary Table S2: Putative identifications for the ten highest absolute ranking mass bins in principal components analysis. Unabridged putative identifications for the ten highest ranking loadings of both components of both principal components analyses (using both positive and negative %TIC). The identity of the compounds given are derived from matching observed masses with the KEGG database (Kanehisa Laboratories, 2017). Blank cells indicate that no match was found.

Detection mode	Component	Mass bin	Ranking	Loading	Metabolite
Negative	PC1	268.4	1	-0.32	
		282.4	2	-0.27	
		267.4	3	-0.19	
		264.6	4	0.17	
		292.6	5	0.16	
		324.6	6	0.15	
		576.4	7	-0.15	Phosphoribosyl-formimino-AICAR-phosphate
		192.4	8	-0.14	
		283.4	9	-0.13	
		269.4	10	-0.13	Estrone
	PC2	282.4	1	-0.46	
		192.6	2	-0.24	
		576.4	3	-0.21	Phosphoribosyl-formimino-AICAR-phosphate
		268.4	4	-0.20	
		300.4	5	0.14	
		284.4	6	0.13	
		242.4	7	0.13	
		254.4	8	0.11	
		577.4	9	-0.11	
		590.4	10	-0.11	
Positive	PC1	285	1	-0.42	Delta3-Isopentenyl diphosphate
					Delta2-Isopentenyl diphosphate
					Isopimpinellin
					Pimpinellin
					D-Mannitol 1-phosphate
					Decursinol
					Marmesin
					Acacetin
					Xanthosine
					Octopine
		381	2	0.29	Trehalose
					alpha-D-glucopyranose
					Nigerose
					Sucrose
					Laminaribiose
					Rutinose
					Maltose
					Melebiose
					Sophorose
					Pantetheine 4'-phosphate
					Cellobiose
					Epimelibiose
					Galactinol

					Gentiobiose
					Isomaltose
		266	3	0.26	alpha-D-Glutamyl phosphate
**Detection mode**	**Component**	**Mass bin**	**Ranking**	**Loading**	**Metabolite**
**Positive**	PC1	266	3	0.26	L-Glutamate 5-phosphate
					L-Arogenate
					Deoxycytidine (dC)
					Cytidine (C)
		447.2	4	-0.24	Ginkgolide A
					Nodakenin
					Adifoline
					Rutarin
					Bleekerine
					Vernoflexuoside
					6,8-Diprenylnarigenin
					Abyssinone V
					Cascarillin
					Dimethamine
		593.2	5	-0.22	
		615.2	6	-0.20	
		222	7	0.20	O-Phospho-L-homoserine
					Isowillardiine
					Willardiine
					L-Normetanephrine
					L-Histidinol phosphate
					N-acetyl-D-galactosamine
					N-acetyl-D-glucosamine
					N-acetyl-D-mannosamine
		286	8	-0.18	Pyridoxal phosphate
					Pyridoxine phosphate
					Linamarin
		594.2	9	-0.14	
		447	10	-0.13	CDP-ethanolamine
					Khellol glucoside
	PC2	266	1	-0.27	*See above (repeated)*
		631.2	2	-0.25	Diosmin
					Reserpine
		381	3	0.22	*See above (repeated)*
		222	4	-0.22	*See above (repeated)*
		285	5	-0.20	*See above (repeated)*
		417	6	0.19	
		365.2	7	0.16	Trehalose
					alpha-D-glucopyranose
					Nigerose
					Sucrose
					Laminaribiose
					Rutinose
					Maltose
					Melebiose
					Sophorose
					Cellobiose

Detection mode	Component	Mass bin	Ranking	Loading	Metabolite
					Epimelibiose
					Galactinol
					Gentiobiose
Positive	PC2	365.2	7	0.16	Isomaltose
					Ajmaline
					Gibberellin A44
					Xanthosine 5'-phosphate (XMP)
		631	8	-0.15	
		411	9	-0.15	2'-Deoxyuridine 5'-diphosphate (dUDP)
		184	10	0.12	Selenophosphate
					L-2-aminoadipic acid
					O-Acetyl-L-homoserine
					2-Ketoglutaramate
					L-Normetanephrine

Supplementary Table S3: Results of a Bonferroni-corrected Dunn's multiple comparison test of specific leaf area between all pairwise combinations of sites. White cells contain the test statistic for that comparison, z, while the grey values are calculated p values. Statistically significant comparisons ($p < 0.05$) are indicated by green cells.

	BOVHL	CLWGW	HARDY	HNTCP	ROUDS
CLWGW	-0.66	-	-	-	-
	1.00				
HARDY	0.90	1.56	-	-	-
	1.00	*0.90*			
HNTCP	-0.13	0.59	-1.18	-	-
	1.00	*1.00*	*1.00*		
ROUDS	**2.79**	**3.27**	2.32	**3.36**	-
	0.04	***0.01***	*0.15*	***0.01***	
SWNTN	1.43	2.04	0.61	1.79	-1.81
	1.00	*0.31*	*1.00*	*0.55*	*0.53*
